# Exploring Older Adult Cancer Survivors’ Digital Information Needs: Qualitative Pilot Study

**DOI:** 10.2196/59391

**Published:** 2025-02-27

**Authors:** Lorelei Newton, Helen Monkman, Claire Fullerton

**Affiliations:** 1 School of Nursing, University of Victoria, PO Box 1700 STN CSC, Victoria, BC, V8W 2Y2, Canada, 1 250-721-6462, 1 250-721-6231; 2 School of Health Information Science, University of Victoria, Victoria, BC, Canada

**Keywords:** older adults, cancer survivors, digital health literacy, digital health technologies, aging, qualitative, pilot study, semistructured interview

## Abstract

**Background:**

Older adults (aged >65 years) are disproportionately affected by cancer at a time when Canadians are surviving cancer in an unprecedented fashion. Contrary to persistent ageist assumptions, not only do the majority of older adult cancer survivors use digital health technologies (DHTs) regularly, such technologies also serve as important sources of their health information. Although older adults’ transition to cancer survivorship is connected to the availability and provision of relevant and reliable information, little evidence exists as to how they use DHTs to supplement their understanding of their unique situation to manage, and make decisions about, their ongoing cancer-related concerns.

**Objective:**

This pilot study, which examined older adult cancer survivors’ use of DHTs, was conducted to support a larger study designed to explore how digital health literacy dimensions might affect the management of cancer survivorship sequelae. Understanding DHT use is also an important consideration for digital health literacy. Thus, we sought to investigate older adult cancer survivors’ perceptions of DHTs in the context of accessing information about their health, health care systems, and health care providers.

**Methods:**

A qualitative pilot study, which involved semistructured interviews with older adult cancer survivors (N=5), was conducted to explore how participants interacted with, accessed, and searched for information, as well as how DHT use related to their cancer survivorship. Institutional ethics approval (#21‐0421) was obtained. Interpretive description inquiry—a practice-based approach suitable for generating applied knowledge—supported exploration of the research question. Thematic analysis was used to examine the transcripts for patterns of meaning (themes).

**Results:**

Assessing the credibility of digital information remains challenging for older adult cancer survivors. Identified benefits of DHTs included improved access to meet health information needs, older adult cancer survivors feeling empowered to make informed decisions regarding their health trajectory, and the ability to connect with interdisciplinary teams for care continuity. Additionally, participants described feeling disconnected when DHTs seemed to be used as substitutes for human interaction. The results of this pilot study were used to create 12 additional questions to supplement a digital health literacy survey, through which we will seek a more fulsome account of the relationship between digital health literacy and DHTs for older adult cancer survivors.

**Conclusions:**

Overall, this pilot study confirmed the utility of DHTs in enhancing the connection of older adult cancer survivors to their health care needs. Importantly, this connection exists on a continuum, and providing greater access to technologies, in combination with human support, leads to feelings of empowerment. DHTs are an important aspect of contemporary health care; yet, these technologies must be seen as complementary and not as replacements for human interaction. Otherwise, we risk dehumanizing patients and disconnecting them from the care that they need and deserve.

## Introduction

Older adults (aged >65 years) are the fastest-growing segment of the Canadian population with unprecedented cancer survivorship [1]. Technology has become increasingly important, with older adult cancer survivors reporting that a significant proportion of their health care information is gathered from digital sources [2]. Despite such availability, finding and accessing information during transitions to survivorship are challenging [3]. Access to information plays a vital role in improving cancer survivors’ health outcomes and quality of life. Although older adult cancer survivors may have unique needs, there is little research examining the extent of digital health technology (DHT) use to support their health. DHTs “use computing platforms, connectivity, software, and sensors for health care and related uses” and include telemedicine, wearable devices, educational resources, remote patient monitoring, etc [4].

With the increasing reliance on DHTs [5], a consistent concern for older adult cancer survivors is interacting with health care systems digitally [6]. Contrary to persistent ageist assumptions, not only do 95% of this group use DHTs regularly, DHTs are substantial sources of their health information [7]. Understanding DHT use is also an important consideration for digital health literacy (DHL). DHL is the capacity “to acquire, process, communicate, and understand health information and services, make effective health decisions, and promote and improve individual and collective health in the context of the use of digital information and technologies” [7]. This pilot study, which examined older adult cancer survivors’ use of DHTs, was conducted to support a larger study designed to explore how DHL dimensions might affect the management of cancer survivorship sequelae.

## Methods

### Ethical Considerations

The pilot study reporting was guided by the COREQ (Consolidated Criteria for Reporting Qualitative Research) checklist (Checklist 1) [8]. Institutional ethics approval (#21‐0421) was obtained from the University of Victoria, and informed consent was secured in advance of conducting interviews. Confidentiality was maintained throughout the research process, and all transcripts were deidentified prior to analysis, with data stored in password-protected files. Participants were given a CAD $25 (US $17.53) gift card.

### Recruitment

Participants were recruited through the social media platforms of local eldercare and retirement groups, and recruitment consisted of 3 recruitment posters that were posted weekly in attempts to elicit diverse responses. As only 2 participants responded to those efforts, an additional 3 participants were recruited, using the snowball method by way of the initial 2 participants’ social networks. The inclusion criteria included being aged 65 years or older, speaking English, having completed cancer treatment within the past 5 years, and being a resident of British Columbia, Canada.

### Data Collection

Semistructured, 90-minute, individual interviews were conducted over a secure videoconferencing platform. Interview questions investigated how participants interacted with, accessed, and searched for information, as well as DHT use related to their cancer survivorship (Textbox 1). The audio-recorded interviews were transcribed and deidentified by the research associate (CF), with two researchers (LN and HM) reviewing for accuracy, prior to analysis. Participants were offered an opportunity to review their transcript for accuracy.

Textbox 1. **Interview questions. Research questions asked to the participants aimed to identify how older adults use digital health tools to interact with, access, and search for information related to their health care and cancer survivorship.**

**Interview questions**

1. What information resource(s) did you use most often?
2. How did you manage multiple information resources?
3. How did you determine the information was credible?
4. Knowing what you know now, what information do you wish you had access to?
5. What questions did you have around your transition from treatment to survivorship?
6. What questions were the most difficult to find answers to?
7. What have you found difficult to understand about this transition?
8. What do you like/dislike about using digital health tools?
9. What makes it easy/difficult for you to use digital health tools?
10. Now that you have completed the eHealth literacy survey, and after thinking about the questions already asked, is there anything else you would like to tell us?

### Reflexivity, Interpretive Description, and Thematic Analysis

All interviews were conducted by 2 PhD-prepared assistant professors (LN and HM) and an undergraduate student, who was included as a research associate (CF). The lead authors have experience with qualitative research; are from the Global North; and identify as cisgender, White, female individuals who endeavor to be reflexive on positionality and perspectives throughout the research process. Interpretive description inquiry—a practice-based approach suitable for generating applied knowledge—supported exploration of the research question [9]. Thematic analysis was used to examine the transcripts for patterns of meaning (themes) [10]. The researchers convened after independently reviewing the first transcript to ensure congruence. As the researchers became familiar with the data and generated initial codes, themes were reviewed together. Finally, the researchers agreed that the themes accurately reflected participants’ responses.

## Results

### Participants

A total of 5 older adult cancer survivors (4 women and 1 man), with an average age of 69 (SD 2.06) years, completed interviews. All 5 had postsecondary education and stated that they accessed DHTs daily. All participants had completed cancer treatment within the past 5 years and represented different geographic areas of the province. Participants were not known to the researchers prior to the interviews; however, researcher information and an overview of the research project were included in the informed consent process. Time and an opportunity for participants to ask questions were provided prior to the interviews. Additionally, during the informed consent process, participants were assured that their data were private and were not to be shared publicly. Thus, supporting data are not available publicly.

### Themes

Thematic analysis [10] highlighted the following three main themes: access, empowerment, and connection.

#### Access

All participants agreed that access to DHTs is crucial for health care purposes and for facilitating health care needs with minimal disruption to routines. Distinguishing between access and accessibility is essential; despite owning digital devices and feeling comfortable with using them, 3 participants had no interest in or did not enjoy using digital devices for health care. However, 1 participant expressed how DHTs “were very helpful” and that they “used them for follow up with therapists and counselors.” Such access allowed health care needs, such as education and remote connection to health care professionals (HCPs), to be addressed conveniently.

#### Empowerment

All participants associated DHT with feelings of empowerment, that is, being involved in their health decisions and being in control of their information (including access). All participants discussed the autonomy provided by DHTs. Participants explained feeling empowered by being in control, by accessing education, and by the ability to book appointment times. One participant said, “You can get all the information you need…I never cease to be amazed at what technology can do.” Another participant expressed their satisfaction with DHTs, stating “I don’t have to track it down; everything is just right there.” As such, participants found that independently booking appointments, retrieving educational materials, and reviewing available medical records (eg, laboratory results) improved their sense of empowerment.

#### Connection

Participants described how DHTs enhanced connection. One participant stated that DHTs “allow for discussion that healthcare professionals wouldn’t [otherwise] have time [to start].” A second participant echoed this, saying “When I was really sick, my doctor called me every day.” This web-based connection was especially important in instances when participants were too ill to commute but not ill enough to be admitted to a hospital. In other cases, participants discovered community support through online groups. For example, one participant found videoconferencing with a survivorship group very helpful. Another participant stated, “I remember [medication] affected emotions; nothing could prepare you for that…My doctor and counselor didn’t really talk about it...going online and finding that other people are having the same impact was very comforting.” In this case, the patient found a sense of community through shared experiences on the web.

Paradoxically, participants also described experiences where human connection was absent. One participant shared an example of when an HCP noticed and addressed a physical concern as they were leaving an in-person appointment. This participant felt that this would never have been noticed in a web-based meeting. Another key observation was how intonation of voice, facial expressions, and body language of HCPs were difficult to discern and negatively impacted the participants’ interpretations of interpersonal communication. One participant expressed frustration with “stereotypes placed on older people.” Another stated, “People tend to treat everyone over the age of 65 like they are homogenous.” Participants expressed annoyance with HCPs often overexplaining technology or assuming a lack of understanding of how to access DHTs when the participants felt that they could complete the task independently. The participants also pointed out instances in which HCPs lacked knowledge about the DHTs they expected patients to use.

### Key Learnings to Inform Future Work

**Figure 1.**
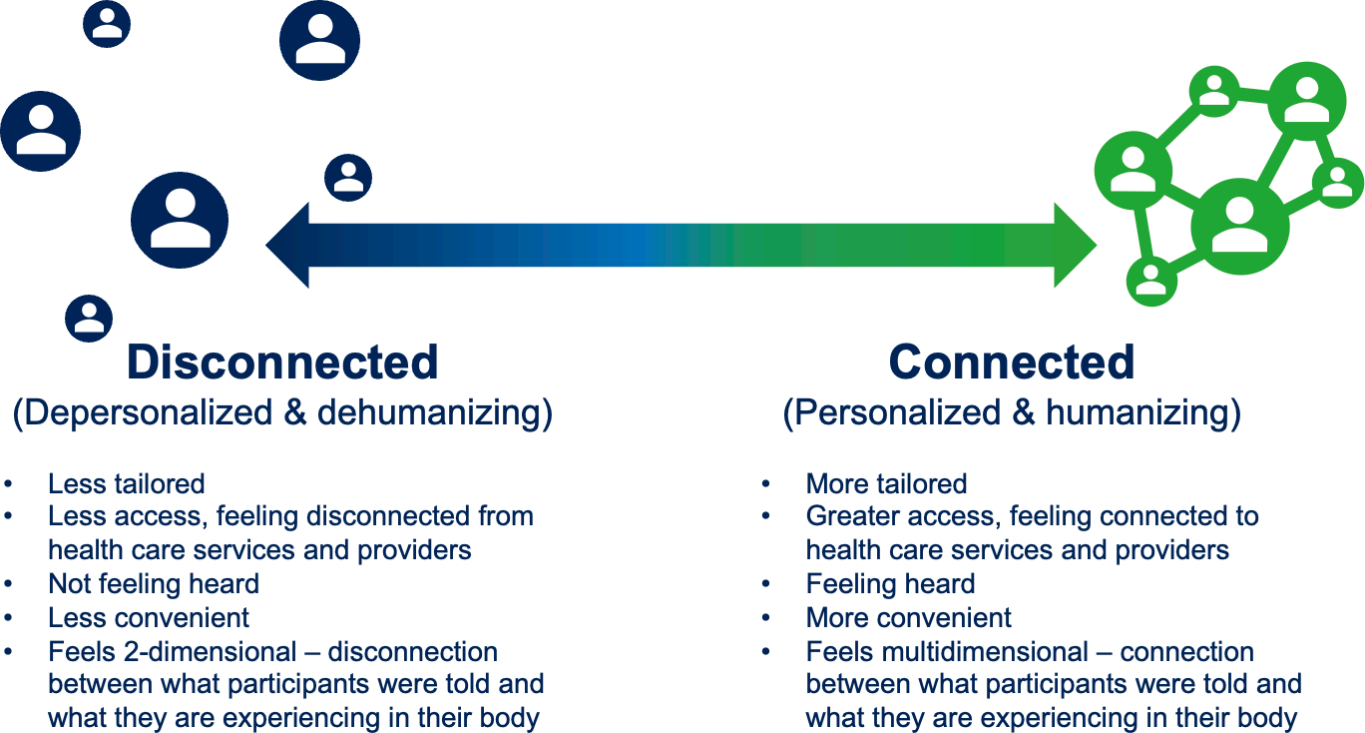
Connection continuum.

Participants reported that DHTs can facilitate access and empowerment. Participants’ experiences also pointed to how DHTs can either foster connection or create barriers to human connection. That is, they described their feelings of connection as along a continuum from feeling disconnected from health care services to feeling connected with health care services (Figure 1). Participants outlined items that contribute to disconnection, such as the feeling of not being heard, services that are less tailored to their situation, and less accessibility. In contrast, participants further described feelings of greater connection with items such as greater access, the feeling of being heard by HCPs, and congruence between information received and what they are experiencing in their body. The results of this pilot study were used to create 12 additional questions to supplement a DHL questionnaire (Textbox 2) for the next phase of the project [11], in which we will seek a more fulsome account of the relationship between DHL and DHTs for older adult cancer survivors.

Textbox 2. **Questions to be included in the eHealth literacy survey. After completing the interviews, the following questions, based on the insights from pilot study participants, were created to address additional areas of importance in the digital health literacy survey.**

**Questions to be included in the eHealth literacy survey**

1. To support your survivorship, what information did you get using digital health tools?
2. Has the COVID-19 pandemic changed the way you think about digital health tools?
3. What digital health tools (apps, websites, wearables) did you use to support health and survivorship and how useful were they?
4. Did you join any support groups for people who have finished cancer treatment?
5. How often do you use digital health tools?
6. Is there anything you like about using digital health tools?
7. Is there anything you dislike about using digital health tools?
8. Is there anything you dislike about digital health tools as a part of health care?
9. If you are frustrated using digital health tools, how did you get through this frustration?
10. Where do you go if you need help with digital health tools?
11. In the past year, have you connected with a health care professional digitally?
12. What recommendations do you have about digital health tools for health professionals and organizations?

## Discussion

### Principal Findings

The participants in this pilot study confirmed some of the benefits of using DHTs for older adults’ cancer survivorship; they can learn about their condition, connect with interdisciplinary teams for continuity of care, find connections and community support, and make educated and informed decisions regarding their health trajectory in survivorship. These findings are congruent with other studies, in which older adult cancer survivors expressed preferences for in-person visits and personalized telehealth visits [12], described how using DHTs could provide a sense of autonomy (ie, by allowing them to be actively involved with their health care) [2], and appreciated access to technology while maintaining a strong preference to be listened to with basic respect [13]. However, the propensity of health care systems to use DHTs as substitutes for human interactions to increase cost-effectiveness and efficiency can counteract those benefits. Indeed, given the existing structural ageism inherent in contemporary health care, there is a real danger of amplifying ageist processes, which can result in care that does not account for the intersection of normal aging and cancer survivorship [13]. Further, with the current lack of accounting for the burdens of navigating challenging cancer care systems, DHTs can either escalate feelings of disconnection or provide opportunities for connection and reconnection. Re-establishing the patient at the center of care and leveraging the humanity possible in DHTs are crucial; to continue to do otherwise will ultimately lead to a sense of disengagement. DHTs are an important aspect of contemporary health care; yet, these technologies cannot replace HCP contact, or we risk dehumanizing patients and disconnecting them from the care that they need and deserve. By using DHTs compassionately and strategically for the ongoing care of older adult cancer survivors, HCPs can support this group along a continuum from feeling disconnected from health care services to feeling connected with health care services. Thus, it is imperative to determine the conditions under which DHTs complement health care and enhance rather than impair connection.

### Limitations

Despite providing insights to augment the future survey, having only 5 participants inherently limits the scope and transferability of these findings. Further, although the participants represented geographic diversity, all identified as White, spoke English, and had postsecondary education; thus, this small, nonrepresentative sample may have reduced the richness of the data and the ability to achieve data saturation. Questions regarding diversity will also be added to the upcoming survey.

### Conclusion

Overall, this pilot study confirmed the utility of DHTs in enhancing the connection of older adult cancer survivors to health care. Importantly, this connection exists on a continuum, and providing greater access to DHTs, in combination with human support, leads to feelings of empowerment. We are confident that applying these findings to further research will illuminate best practices for supporting older adult cancer survivors to optimize their cancer-free years.

## Supplementary material

10.2196/59391 Checklist 1 COREQ (Consolidated Criteria for Reporting Qualitative Research) checklist.

## Abbreviations

COREQ: Consolidated Criteria for Reporting Qualitative Research
DHL: digital health literacy
DHT: digital health technology
HCP: health care professional
